# Development and Reliability Evaluation of the Movement Rating Instrument for Virtual Reality Video Game Play

**DOI:** 10.2196/games.5528

**Published:** 2016-06-01

**Authors:** Danielle Levac, Joanna Nawrotek, Emilie Deschenes, Tia Giguere, Julie Serafin, Martin Bilodeau, Heidi Sveistrup

**Affiliations:** ^1^Northeastern UniversityDepartment of Physical Therapy, Movement and Rehabilitation SciencesBoston, MAUnited States; ^2^University of OttawaSchool of Rehabilitation SciencesOttawa, ONCanada; ^3^University of OttawaSchool of Human KineticsOttawa, ONCanada; ^4^Bruyere Research InstituteOttawa, ONCanada

**Keywords:** active video games, virtual reality, physical therapy, movement, reliability

## Abstract

**Background:**

Virtual reality active video games are increasingly popular physical therapy interventions for children with cerebral palsy. However, physical therapists require educational resources to support decision making about game selection to match individual patient goals. Quantifying the movements elicited during virtual reality active video game play can inform individualized game selection in pediatric rehabilitation.

**Objective:**

The objectives of this study were to develop and evaluate the feasibility and reliability of the Movement Rating Instrument for Virtual Reality Game Play (MRI-VRGP).

**Methods:**

Item generation occurred through an iterative process of literature review and sample videotape viewing. The MRI-VRGP includes 25 items quantifying upper extremity, lower extremity, and total body movements. A total of 176 videotaped 90-second game play sessions involving 7 typically developing children and 4 children with cerebral palsy were rated by 3 raters trained in MRI-VRGP use. Children played 8 games on 2 virtual reality and active video game systems. Intraclass correlation coefficients (ICCs) determined intra-rater and interrater reliability.

**Results:**

Excellent intrarater reliability was evidenced by ICCs of >0.75 for 17 of the 25 items across the 3 raters. Interrater reliability estimates were less precise. Excellent interrater reliability was achieved for far reach upper extremity movements (ICC=0.92 [for right and ICC=0.90 for left) and for squat (ICC=0.80) and jump items (ICC=0.99), with 9 items achieving ICCs of >0.70, 12 items achieving ICCs of between 0.40 and 0.70, and 4 items achieving poor reliability (close-reach upper extremity-ICC=0.14 for right and ICC=0.07 for left) and single-leg stance (ICC=0.55 for right and ICC=0.27 for left).

**Conclusions:**

Poor video quality, differing item interpretations between raters, and difficulty quantifying the high-speed movements involved in game play affected reliability. With item definition clarification and further psychometric property evaluation, the MRI-VRGP could inform the content of educational resources for therapists by ranking games according to frequency and type of elicited body movements.

## Introduction

There is increasing evidence for the potential of virtual reality active video games to challenge balance, promote active movement, and increase energy expenditure in children with cerebral palsy [1-3]. To support the integration of virtual reality and active video games into clinical practice, physical therapists require educational resources that inform decision making about matching virtual reality active video games with differing client needs [4]. A recent survey of Canadian physical and occupational therapists found that 76% reported the need for knowledge translation resources providing information about specific systems and games [5]. These resources can build on existing information, including a classification framework that categorizes systems based on characteristics such as the ability to track therapeutically relevant variables [6], a Nintendo Wii and Wii Fit game analysis table that describes general movement requirements and feedback provided by each game [7], and a systematic assessment of serious games in health care [8]. However, selecting from among the wide variety of games available in off-the-shelf systems such as the Nintendo Wii and Microsoft Kinect can be overwhelming for busy clinicians. Resources that provide more detailed information about each game are required.

Specifically, information about the type and frequency of movements elicited during game play across differing games and systems can inform decisions about which game may be best suited for an individual client’s physical capabilities and rehabilitation needs. For instance, whereas one game might elicit more squats and be ideally suited to strengthening the lower extremities, another game might elicit more upper extremity reaches and be better suited to increasing shoulder range of motion. Distinguishing between games that elicit movements within the base of support (BOS; eg, trunk leans) and those that elicit movements outside of it (eg, steps) is important from a therapeutic perspective because this categorization relates to different levels of functioning. For example, a therapeutic goal may be to enhance energy expenditure, in which case, the number of steps outside of the BOS, squats, and jumps are relevant. A different goal may be to increase weight shifting to one side of the body, and trunk leans or far reaches within the BOS may be important in that case. In addition, understanding the similarities between games across different systems can inform therapist decisions about progressing use of virtual reality active video games from the clinic to the home.

A valid and reliable instrument quantifying the type and frequency of body movements elicited during game play is required. The objectives of this study were to: (1) develop the Movement Rating Instrument for Virtual Reality Game Play (MRI-VRGP); (2) examine the feasibility of using the MRI-VRGP to rate videotaped game play sessions; and (3) evaluate the instrument’s inter-rater and intrarater reliability.

## Methods

### Study Design

A measurement study design was used to develop the MRI-VRGP and evaluate its feasibility and reliability.

### Instrument Development

An iterative process of item generation and refinement involving the study authors was undertaken by 3 researchers (2 of whom are also physical therapists) and 4 physical therapy students. The authors began by undertaking a literature search to identify existing instruments to quantify body movements elicited during virtual reality game play. Finding none, we discussed the movement characteristics that might be relevant to physical therapists interested in differentiating between virtual reality active video games. We then watched sample videotapes of typically developing children playing virtual reality active video games to generate an initial list of items. A series of 4 meetings was undertaken. After each meeting, students and investigators went back to practice videos and discussed refinements to the items. The final instrument is shown in Multimedia Appendix 1.

The MRI-VRGP items represent 12 upper extremity, lower extremity, or full-body movements involved in game play. A rater repeatedly views a videotaped game play session and records the frequency with which each movement is observed. Upper extremity movements are identified as unilateral or bilateral (occurring simultaneously) and separated into close reaches and far reaches. Full body weight shifts that occur inside the BOS are identified by direction (anterior, left, or right), and movements that occur outside the BOS such as lower extremity steps are identified by the limb (left or right) and direction (anterior, posterior, lateral, and crossing midline). Full-body movements of squats and jumps are recorded. Rater training materials with operational definitions for each item (summarized in Table 1) were developed in conjunction with instrument creation.

**Table 1:** Summary of operational movement definitions.

MRI-VRGP items^a^	Definitions
Close reach	Flexion of elbow >90 **°**
Far reach	Flexion of elbow <90 **°**
Front weight shift inside base of support	Front inclination >10° without bending knees
Side weight shift inside base of support	Side inclination >10° with or without bending knees
Single leg stance	Single leg stance > 2 seconds
Front weight shift outside base of support	Forward step
Side weight shift outside base of support	Side step
Back weight shift outside base of support	Backward step
Cross midline weight shift outside base of support	Forward or backward step that crosses the midline
Squat	Knee bend with forward inclination of trunk
Jump	Clearing ground with both feet

^a^MRI-VRGP: Movement Rating Instrument for Virtual Reality Game Play.

### Reliability and Feasibility Evaluation

Three physical therapist student raters who had participated in instrument creation underwent a training process in which they and the researchers each rated 3 sample videotapes and met to discuss their results, coming to consensus about each item on each videotape. The students subsequently rated 176 videos. Each rater watched each video at least 3 times, focusing on upper extremity, lower extremity inside the BOS, and lower extremity movements outside the BOS movements separately on each viewing. Video pauses and playbacks were encouraged to maximize the quality of scoring. For intrarater reliability evaluations, each student re-rated 58 videos, a minimum of 1 week after the initial rating. The total time period of rating was approximately 2.5 months. A fourth physical therapy student determined the rating schedules. Raters were blinded to the game that the child was playing and whether the child was typically developing or had cerebral palsy. After completion of the MRI-VRGP for each video, the rater completed a feasibility evaluation involving questions determined by study authors. A 5-cm visual analog scale was used to quantify rating difficulty, with the anchor at 0 cm described as “Easy” and the anchor at 5 cm described as “Difficult.” A similar scale was used to quantify rater confidence, with anchors on “low” and “high.” Raters recorded the time taken to watch the video and complete the MRI-VRGP. Raters provided comments if required to identify video-specific rating difficulties.

### Videotaping Participants

The videotapes used in this study were recorded in the context of our previous study exploring energy expenditure during virtual reality active video game play in typically developing children and children with cerebral palsy (Levac D, PhD, 2014 unpublished data). Children and parents provided informed assent or consent for videotaping. Overall, 176 prerecorded videotapes of 11 children that were each 90 seconds long, playing the games against a standardized green backdrop were used. The videotapes involved 4 children (3 girls, 1 boy) with cerebral palsy classified at Gross Motor Function Classification System Level 1 (mean age 12.75 years, standard deviation (SD) 2.87 years) and 7 typically developing children (5 girls, 2 boys; mean age 12.86 years SD: 2.97 years). The participants reported minimal exposure (<5 hours) to virtual reality active video games before the study.

Study participants played 8 90-second games on 2 systems: the Interactive Rehabilitation and Exercise System (IREX; GestureTek Health; www.gesturetek.com) and the Microsoft Kinect for Xbox 360. The IREX and the Kinect use similar motion capture technology; in the IREX, the user’s image is embedded in the virtual environment where they can interact with virtual objects [9], whereas Kinect games involve full-body movement represented onscreen by an avatar. The 8 games (IREX: Snowboarding, Shark Bait, Zebra Crossing, Soccer; Kinect: Space Pop, Reflex Ridge, River Rush, and 20,000 Leaks) were chosen to represent the range of movement possibilities across games on each system. Each game was played at its easiest difficulty level.

### Statistical Analysis

Analyses were conducted using Statistical Package for the Social Sciences (SPSS; version 21.0). Intrarater and interrater reliability were determined for the total score, category totals, and for each item of the MRI-VRGP. Intraclass correlation coefficient (ICC; type [1,0k] random effects model) and associated 95% CI were calculated. Traditionally used comparators of < 0.40 as low, 0.4 to 0.74 as moderate, and 0.75 and higher as good for ICCs were used [10]. An ICC > 0.75 with a 95% CI lower bound of 0.60 was set a priori as acceptable for each item. Descriptive statistics summarize time, ease, and confidence ratings across raters. Analysis of variances compare differences in time, ease, and confidence ratings between the 3 raters.

## Results

### Reliability

Tables 2 and 3 summarize intrarater ICCs and 95% confidence intervals as well as the range of observed frequencies for each item per rater. ICCs for rater 1 ranged from 0 to 0.99, rater 2 from 0.54 to 1, and rater 3 from 0.06 to 1. For each rater, far-reach upper extremity movements and full-body jump movements had the highest ICCs, whereas close-reach upper extremity movements and lower extremity movements outside of BOS had the lowest ICCs.

**Table 2:** Intrarater intraclass correlation coefficients of upper extremity movements.

Movement Rating Instrument Items		Rater 1				Rater 2				Rater 3			
		Intrarater ICC	CI	Range	Intrarater ICC	CI	Range		Intrarater ICC	CI	Range
**Upper extremity movements**															
	Close-reach right arm	0.967	0.945-0.98	0-45	0.976	0.985-0.96	0-61		0.831	0.732-0.896	0-5
	Far-reach right arm	0.993	0.988 to −996)	0-202	0.987	0.978-0.992	0-144		0.977	0.962-0.986	0-214
	Total movements right arm	0.992	0.987-0.995	0-202	0.99	0.983-0.994	0-195		0.977	0.962-0.986	0-214
	Close-reach left arm	0.989	0.981-0.993	0-46	0.964	0.978-0.94	0-56		0.765	0.635-0.853	0-4
	Far-reach left arm	0.995	0.991-0.997	0-189	0.987	0.977-0.992	0-133		0.977	0.962-0.986	0-215
	Total movements left arm	0.995	0.991-0.997	0-189	0.986	0.976-0.992	0-182		0.977	0.962-0.986	0-215
	Close-reach bilateral	0	−0.255 to 0.256	0-9	0.86	0.775-0.914	0-23		0.064	0.192-0.312	0-7
	Far-reach bilateral	0.987	0.962-0.987	0-54	0.983	0.971-0.99	0-42		0.99	0.984-0.994	0-64
	Total bilateral movements	0.977	0.962-0.986	0-54	0.985	0.975-0.991	0-51		0.983	0.971-0.99	0-64

**Table 3:** Intrarater intraclass correlation coefficients (ICC) of lower extremity movements.

Movement Rating Instrument Items		Rater 1			Rater 2				Rater 3		
		Intrarater ICC	CI	Range	Intrarater ICC		CI	Range	Intrarater ICC		CI	Range
**Lower extremity movements**						
									
	Front lean	0.684	0.52-0.8	0-10	0.703		0.548-0.812	0-12	0.915		0.861-0.948	0-10
	Side lean right	0.863	0.78-0.917	0-20	0.827		0.726-0.893	0-16	0.915		0.862-0.949	0-18
	Side lean left	0.926	0.878-0.955	0-18	0.786		0.665-0.867	0-16	0.915		0.862-0.949	0-13
	Single-leg stance right leg	0.973	0.955-0.984	0-12	0.856		0.77-0.912	0-5	0.643		0.466-0.771	0-12
	Single-leg stance left leg	0.957	0.929-0.974	0-8	1		1	0-6	0.622		0.439-0.757	0-5
	Front step right leg	0.956	0.927-0.974	0-34	0.94		0.901-0.964)	0-39	0.921		0.871-0.952	0-32
	Side step right leg	0.898	0.834-0.938	0-39	0.548		0.343-0.704	0-25	0.932		0.888-0.959	0-46
	Back step right leg	0.956	0.927-0.974	0-27	0.936		0.895-0.961	0-31	0.94		0.902-0.964	0-28
	Cross midline right leg	0.43	0.196-0.617	0-11	0.983		0.971-0.99	0-8	0.766		0.636-0.853	0-5
	Front step left leg	0.957	0.929-0.974	0-35	0.962		0.937-0.977	0-39	0.943		0.906-0.966	0-32
	Side step left leg	0.939	0.9-0.963	0-42	0.541		0.334-0.698	0-30	0.947		0.913-0.968	0-49
	Back step left leg	0.963	0.939-0.978	0-35	0.899		0.836-0.938	0-34	0.929		0.884-0.957	0-30
	Cross midline left leg	0.953	0.923-0.972	0-6	0.962		0.936-0.977	0-13	0.867		0.787-0.919	0-11
	Squat	0.98	0.967-0.988	0-24	0.933		0.889-0.959	0-45	0.918		0.867-0.951	0-23
	Jump	0.99	0.982-0.994	0-62	0.995		0.992-0.997	0-57	0.997		0.995-0.998	0-56

Table 4 summarizes interrater reliability findings for each item. The ICC was high for far-reach bilateral (ICC=0.94) and low for close reach in both upper extremities (ICC=0.07). For full-body and lower extremity movements, the highest ICC was for the jump item (ICC=0.99) and the lowest for single-leg stance left leg (ICC=0.27).

**Table 4:** Interrater intraclass correlation coefficients (ICCs).

Movement Rating Instrument Items		Interrater values					
			Interrater ICC	CI	Mean (SD) Rater 1	Mean (SD) Rater 2	Mean (SD) Rater 3
**Upper extremity movements**						
					
	Close-reach right arm		0.14	0.05-0.24	1.60 (5.08)	3.89 (8.83)	0.18 (0.69)
	Far-reach right arm		0.92	0.89-0.94	19.39 (32.88)	21.30 (29.77)	20.14 (29.74)
	Total movements right arm		0.93	0.91-0.95	20.99 (33.35)	25.19 (34.25)	20.31 ( 29.78)
	Close-reach left arm		0.07	−0.02 to 0.16	1.00 (4.02)	3.12 (8.15)	0.16 (0.57)
	Far-reach left arm		0.90	0.88-0.92	17.22 (31.09)	19.63 (28.63)	19.06 (29.43)
	Total movements left arm		0.93	0.91-0.94	18.23 (31.35)	22.74 (32.46)	19.22 (29.42)
	Close-reach bilateral		0.10	0.01-0.20	0.15 (0.89)	0.74 (2.59)	0.09 (0.57)
	Far-reach bilateral		0.94	0.93-0.96	5.35 (9.79)	5.12 (9.38)	5.52 (9.66)
	Total bilateral movements		0.93	0.92-0.95	5.49 (9.98)	5.80 (10.81)	5.61 (9.67)
**Lower extremity movements**						
		
	Front lean		0.62	0.54-0.69	0.68 (1.50)	0.52 (1.41)	0.39 (1.35)
	Side lean right		0.72	0.65-0.77	2.39 (3.44)	2.57 (3.80)	1.68 (3.09)
	Side lean left		0.63	0.56-0.70	2.14 (3.18)	2.35 (3.42)	1.43 (2.64)
	Single-leg stance right leg		0.55	0.46-0.62	0.28 (1.24)	0.11 (0.56)	0.40 (1.38)
	Single-leg stance left leg		0.27	0.18-0.37	0.16 (0.88)	0.11 (0.60)	0.20 (0.61)
	Front step right leg		0.60	0.52-0.67	3.04 (4.86)	10.85 (7.79)	2.69 (4.94)
	Side step right leg		0.57	0.49-0.64	13.80 (9.24)	7.66 (5.84)	17.59 (9.70)
	Back step Right Leg		0.67	0.60-0.73	2.61 (4.22)	7.94 (7.02)	2.60 (4.23)
	Cross midline right leg		0.68	0.61-0.74	0.35 (1.19)	0.55 (1.41)	0.38 (0.94)
	Front step left leg		0.55	0.47-0.63	3.26 (4.86)	10.46 (7.94)	2.85 (4.85)
	Side step left leg		0.54	0.46-0.62	14.02 (9.30)	7.74 (6.12)	17.69 (9.80)
	Back step left leg		0.73	0.67-0.79	2.68 (4.41)	7.26 (6.30)	2.35 (4.04)
	Cross midline left leg		0.70	0.64-0.76	0.35 (0.89)	0.68 (1.64)	0.32 (1.04)
	Squat		0.80	0.76-0.85	5.00 (6.28)	7.96 (9.70)	5.01 (6.18)
	Jump		0.99	0.98-0.99	7.48 (11.94)	7.72 (12.14)	7.49 (11.57)

### Feasibility

The mean (SD) difficulty of rating score was 1.89 (0.26) of 5. The mean (SD) confidence of rating score was 3.44 (0.24) of 5. Raters took an average of 14.37 (0.77) minutes (range 4-27) per video. There was a significant difference between raters in difficulty ratings (*P* <.001), with rater 3 finding rating to be more easy as compared with raters 1 and 2 finding ratings. There was a significant difference between raters in confidence ratings (*P* <.001) with rater 1 being less confident than raters 2 and 3. Finally, there was a significant difference in time to rate the videos (*P* <.001), with rater 2 taking more time than rater 1 or 3. Comments on the form indicated that raters often were not able to visualize the child’s legs or feet because of camera angle and that videos in which movements were occurring faster and at a higher frequency were more challenging to rate.

## Discussion

Intrarater reliability estimates for each of the 3 raters indicate that individual raters were consistently able to record frequency of 16 of the 25 items in the MRI-VRGP on repeated viewing of a videotaped game play session at a reliability rate of greater than the predetermined ICC of 0.75 and lower bound CI of 0.60. The 8 items with which 1 or more raters had difficulty were front lean, side step right leg, side step left leg, single-leg stance right leg, single-leg stance left leg, close-reach bilateral, and cross midline right leg. The lower bound of CIs stayed well above the targeted range for acceptable reliability, with the exception of those items. However, interrater reliability estimates were less precise, with ICCs ranging from poor to excellent and wider 95% CIs. Despite this lack of precision, 8 items were above the preidentified ICC and CI range for acceptance, 12 items were between 0.40 and 0.74, and only 4 items were <0.40. These 4 most problematic items were upper extremity close reach, total body within the BOS, and lower extremity within and outside BOS items.

The upper extremity items that were problematic across both intrarater and interrater reliability estimates included close reaches (both unilateral—left or right—and bilateral). Lack of clarity in item definitions likely contributed to rater inconsistencies. For example, the distinction between close- and far-reach was defined as an elbow flexion angle of greater than 90°, but, the speed of movements made this angle difficult to determine while watching the video, and ICCs were very low for this item (ranging from 0.07 to 0.14). Although each rater identified differing numbers of close- and far-reaches, total combined arm movements (left, right, and bilateral) had good interrater reliability, indicating that the raters reported similar amounts of total arm movements but that problems arose in distinguishing between “close” and “far.” We had included this distinction between close- and far-reach based on our discussions of the therapeutic relevance of different reach ranges. Therapists might be interested in knowing how often children are required to make a potentially more challenging (ie, in a greater joint range of motion) upper extremity movement. However, confirming with practicing therapists as to the clinical relevance of categorizing upper extremity movements in this way is an important next step in instrument revisions.

With respect to trunk and lower extremity items, single-leg stances, front leans, and side steps were most problematic. Video quality likely impacted difficulties identifying trunk and lower body movements. Front leans are defined as “an isolated movement that cannot precede a step.” Distinguishing leans from steps was problematic because some raters likely included a lean within a step, whereas others may have counted the 2 movements separately. Indeed, in 96 of the 176 videos (54.5%), the camera angle did not allow for the visualization of participants’ feet. This was detrimental when rating items such as weight shifts within the BOS, such as single-leg stance or side steps, where seeing whether the foot lifted off the ground was essential for item scoring. The 2 lower extremity items that achieved good interrater reliability—jumps and squats—are clearly distinguishable movements that can be identified appropriately even without visualizing the feet.

Movement speed and differing game play strategies across children are issues that impacted reliability. Two games in particular on the Xbox Kinect system—Space Pop and Rally Ball—required rapid upper extremity movements. Raters needed to slow down the video speed or pause the video repeatedly. In the Space Pop game, arm movements to simulate flying are needed to “pop” the virtual bubbles. These high-speed movements may have led to interrater differences in counts because movements may have been missed or counted twice. In addition to movement speed, differing game play strategies that enhanced the variation across children playing the same game were observed. Although each game was played at the same difficulty level, individual children chose to focus on different components of the game (eg, choosing to go for all the “coins” in Reflex Ridge by moving their arms or choosing to focus only on body movements that avoided the obstacles). In addition, during the 90 seconds, some children advanced further in the game than others; one game in particular (Rally Ball) required quiet standing while it reset to the previous level if a player was unsuccessful, limiting movement options during this resetting time (approximately 3-5 seconds). Despite controlling for difficulty level and duration of play, children’s game play abilities and their level of success at each game during those 90 seconds resulted in a wide variation of movements that related both to each child’s personal “style” (ie, did they move in a slower, more controlled manner or did they use rapid, flailing movements) and to choice of what to focus on for each game (ie, getting as many points as possible or making as few errors as possible).

From a feasibility perspective, despite these issues, raters found it fairly easy to rate and were fairly confident, although rater 3 found it the most difficult, and rater 2 was the most confident. Interestingly, rater 2, who was the most confident, also had the highest mean rating time. As anticipated, given the protocol requiring a minimum of 3 viewings, rating time was long for such a short video, indicating that raters likely slowed down the video speed and stopped the tapes on a frequent basis while watching and rewatching.

Skjaeret et al [11] were the first to systematically observe movement characteristics of users during videotaped active video game play. Using a 5-point Likert scale, the researchers rated 5 movement characteristics considered relevant to fall prevention exercises in seniors playing 3 virtual reality active video games [11]. Their goal was to inform the design of new virtual reality active video games for this population. Raters also watched each video numerous times to focus solely on a single movement characteristic per viewing. The movement characteristics that they examined included amount of weight shift, temporal variation, step length variation, variation in movement direction, and visual independency [11]. They achieved high interrater reliability across 3 raters (>0.840) for all characteristics. Rating movement characteristics that can be judged in summary after watching a video as opposed to frequency counts of more specific movements may be a method to increase the consistency of observations across raters. For the population of children with cerebral palsy, other global movement characteristics might be more relevant, including cross midline movements and bilateral reaches.

Finally, it is important to consider the amount of error that is acceptable for this type of instrument. The purpose of the MRI-VRGP is to document the frequency of movements elicited during game play. Thus, the magnitude of error that is acceptable for this instrument is greater than would be the case if the purpose was to use it for making decisions about an individual child’s treatment or progress. Given that information obtained through the use of this instrument will be used to inform comparisons between virtual reality active video games and systems, subsequent steps in the instrument evaluation process will focus on determining whether items can be made more general (eg, is the magnitude of reach for arm movements important?) and on better defining each movement that is rated through a validity process.

### Limitations

MRI-VRGP items were established by a small group of researchers and physical therapy students. The research team arrived at the items and their definitions through a literature search of movement characteristics of children with cerebral, energy expenditure related to different virtual reality active video games, viewing of sample videotapes, and clinical understanding of the movements that physical therapists would be interested in when selecting a particular game for a therapy intervention. However, a more formal face and content validity process with additional experts and clinicians would have determined whether the chosen items are representative of what clinicians would like to capture and may have served to clarify the item operational definitions before reliability testing. In addition, involving raters who were not involved in instrument development would have strengthened the findings.

The MRI-VRGP provides clinicians with a simple count of movements but does not include an analysis of movement quality. This may be an issue if therapists are interested in both how often a game elicits a particular movement and the quality of that movement. Moreover, the scale does not quantify whether the player has used potentially unwanted or therapeutically harmful compensations required to achieve a certain movement. For example, the instrument does not distinguish or document whether a child is using shoulder hiking to reach a target above them or using hip circumduction to take a step. It may be important to include a section where the rater can make note of any perceived maladaptive movement patterns during game play. This is particularly important if therapists are using the instrument to inform development of unsupervised home programs. In supervised situations, maladaptive movements can be monitored by the therapist as the child plays the game. Given that this is not possible in supervised exercise, therapists can use these observations to recommend changes to game parameters that might avoid them (eg, recommending that the child play at a lower difficulty level, which may slow down the game and reduce unwanted movements).

Motion analysis systems were once limited to laboratory use, but, the introduction of the Kinect sensor has made markerless motion analysis feasible on a wider scale. How can an observer-rated measure quantifying movement frequency be a useful adjunct to this low-cost kinematic sensor? Reports exploring the psychometric properties of the Kinect sensor to measure movement across a wide variety of populations and tasks are available; accuracy and reliability are inconsistent and dependent on the type and frequency of movement (eg, [12-14]). As evidence continues to emerge to support use of the Kinect sensor for kinematic analysis, the MRI-VRGP could act as an adjunct to quantify movement frequency as the Kinect provides information to therapists that can be used to assess movement quality.

Finally, videotapes of typically developing children and children with cerebral palsy were included in this study. There was a wide range of frequency of movements observed for each of the items, implying sufficient heterogeneity of the measured construct to enable reliability analyses. The 8 games targeted upper extremity and lower extremity movements to different extents. However, the small sample size of participants reduced the precision of the reliability estimates. This first attempt at developing the instrument and evaluating reliability indicated issues of strengths and weaknesses that can be built on in future work.

### Future Recommendations

Given that most items in both intrarater and interrater reliability achieved a minimum of good reliability in this preliminary investigation, further refinements will be undertaken. Subsequent steps include videotaping a greater number of children and youth to use as the basis for adding greater clarity to item definitions. Items will then be put to a Delphi process with pediatric physical therapists to achieve consensus on content and definition. The revised items and definitions will be on the basis of a systematic rater training procedure, involving the new videotapes. Subsequently, psychometric property testing on a larger sample size of typically developing children will be undertaken. If shown to have adequate reliability, therapists could use these numbers as a baseline when making decisions about game use for their clients with cerebral palsy or other diagnoses. The instrument could also be used as a tool to compare movements elicited in different games across different virtual reality active video game systems, adding objective information to include in clinical decision-making tools that help clinicians make decisions about which games to use for different clinical goals. Multiple games from different systems will be included in future work. The result will be a game ranking from most to least elicited movements in each category, allowing clinicians to select the game that elicits the movements most important for an individual child’s rehabilitation needs.

### Conclusions

The MRI-VRGP demonstrated overall good intrarater reliability and moderate interrater reliability. Poor video quality, rater inconsistencies in terms of interpretation of operational movement definitions, and difficulty quantifying movements occurring at high speed contributed to these findings. With subsequent development and psychometric property evaluation, a valid and reliable instrument could be used to provide objective information about movement quantity across different games and systems, contributing to clinical decision-making tools that will inform game selection by clinicians for a broad range of clients.

## Abbreviations

BOS: base of support
ICC: intraclass correlation coefficient
MRI-VRGP: Movement Rating Instrument for Virtual Reality Game Play
