# Supplementary material for: Development and Reliability Evaluation of the Movement Rating Instrument for Virtual Reality Video Game Play
Source: JMIR Serious Games. 2016 Jun 1;4(1):e9. doi: 10.2196/games.5528 (PMC4909974; doi:10.2196/games.5528)
Supplement: Supplementary file 1 [file games_v4i1e9_app1.pdf]

Movement Rating Instrument for Virtual Reality Game Play

Upper Extremity Movements		Right arm	Left arm	Bilateral	Total
	Close reach				
	Far reach				
	Total				
Lower Extremity Movements	Weight shift inside base of support (BoS)				Total
	Front				
		Right leg	Left leg		Total
	Side				
	Single leg stance				
	Total				
	Weight shift outside BoS (step)	Right leg	Left leg		Total
	Front				
	Side				
	Back				
	Cross midline				
	Total				
	Other				Total
	Squat				
	Jump				

Rating this video was very:

Easy — Hard

The confidence I have in my evaluation is:

Low — High
